# Combination of hyperglycaemia and hyperlipidaemia induces endothelial dysfunction: Role of the endothelin and nitric oxide systems

**DOI:** 10.1111/jcmm.15787

**Published:** 2020-12-26

**Authors:** Niroz Abu‐Saleh, Hiba Yaseen, Safa Kinaneh, Mogher Khamaisi, Zaid Abassi

**Affiliations:** ^1^ Department of Physiology Ruth & Bruce Rappaport Faculty of Medicine Technion Haifa Israel; ^2^ Department of Medicine D Rambam Health Care Campus and Ruth & Bruce Rappaport Faculty of Medicine Technion‐IIT Haifa Israel; ^3^ Clinical Research Institute Rambam Health Care Campus Haifa Israel; ^4^ Department of Laboratory Medicine Rambam Health Care Campus Haifa Israel

**Keywords:** endothelial cells, hyperglycaemia, nitric oxide, oxidized‐LDL endothelin

## Abstract

Endothelial dysfunction (ED) is a key feature of diabetes and is a major cause of diabetic vasculopathy. Diabetic patients who also exhibit hyperlipidaemia suffer from accelerated vascular complications. While the deleterious effects of high glucose levels (HG) and hyperlipidaemia alone on ED are well established, the effects of combined hyperlipidaemia and HG have not been thoroughly studied. Therefore, the current study examines whether HG and hyperlipidaemia exert synergistic ED, and explores the mechanisms underlying this phenomenon. We applied multi‐disciplinary approaches including cultured HUVECs and HMEC‐1 as well as knockout mice CByJ.129S7(B6)‐Ldlrtm1Her/J (LDLR^−/−^) to investigate the mechanisms underlying combined HG and hyperlipidaemia‐induced ED. Incremental doses of glucose in the presence or absence of OxLDL were added to HUVECs and HMEC‐1. After 5 days, the status of nitric oxide (NO) and endothelin (ET)‐1 systems as well as their signal transduction were assessed using Western blot, ELISA and immunoreactive staining. The effects of chronic combination of HG and hyperlipidaemia on endothelial integrity and function as well as alterations in circulatory NO and ET‐1 systems were examined in knockout mice LDLR^−/−^ and their wild‐type. HUVEC cells exposed to HG and OxLDL displayed enhanced ET‐1 production, more than HG or OxLDL when added alone. Overproduction of ET‐1 stems from up‐regulation of endothelin converting enzyme (ECE)‐1 as observed under these conditions. In contrast, combination of HG and OxLDL dramatically decreased both total endothelial NO synthase (eNOS) by 60%, and activated eNOS (peNOS) by 80%. Moreover, NRF2 decreased by 42% and its active form (pNRF2) by 56%, as compared to baseline. Likewise, ET_B_ levels decreased by 64% from baseline on endothelial cells. Furthermore, diabetic LDLR^−/−^ mice displayed a higher blood pressure, plasma triglycerides, cholesterol, ET‐1 and NO2/NO3 levels, when compared with normoglycemic LDLR^−/−^ and BALB mice. Combined hyperglycaemia and hyperlipidaemia activates the ET system and attenuates the nitric oxide system with the Nrf2 signalling pathway. These findings suggest that perturbations in these paracrine systems may contribute to ED.

## 1 INTRODUCTION

Atherosclerosis and coronary artery disease (CAD) are a common feature of patients with diabetes mellitus (DM).^1^, ^2^ Although endothelial dysfunction (ED) and poor vascular regeneration/repair have been implicated in the pathogenesis of micro‐ and macrovascular damage characterizing DM,^3^ the mechanisms underlying ED in this clinical setting are poorly characterized.

Endothelial cells secrete numerous autocrine, paracrine and endocrine substances that regulate vascular tone, coagulation, oxidative stress and inflammation.^4^ Under normal conditions, endothelium maintains a negligible oxidative stress and net vasodilation by releasing several vasoactive mediators, such as prostacyclin (PGI2), nitric oxide (NO), Angiotensin II (Ang II), reactive oxygen species (ROS), thromboxane A2 and endothelin‐1 (ET‐1).^4^, ^5^, ^6^, ^7^ The latter is produced preliminarily from big ET‐1 via endothelin converting enzyme (ECE)‐1.^5^ Preservation of the balance between these opposed factors is crucial for normal endothelial function on one hand, whereas imbalanced generation of these substances results in ED and eventually deleterious structural consequences, on the other.^8^ Among the leading causes of ED, one may list dyslipidaemia, hypertension and DM.^9^, ^10^, ^11^ Hallmark feature of these disease states is reduced production of NO^12^ and enhanced generation of ET‐1 and ROS^13^, ^14^, ^15^, ^16^ by the vasculature. Emerging evidence links ED to insulin resistance as the case in DM and obesity.^4^ Although the exact mechanisms underlying ED in DM are unknown, the fact that DM is characterized by hyperglycaemia and hyperlipidaemia, it is appealing to assume that high glucose and lipid levels have direct adverse effects on endothelial functionality,^17^, ^18^, ^19^ and that reduced NO production along accelerated ET‐1 generation plays a pivotal role in the development of ED.

In patients with CAD, oxidized low‐density lipoprotein (OxLDL) is an independent risk factor for future cardiovascular events.^20^ In this context, it has been demonstrated that OxLDL promotes apoptosis in primary human umbilical vein endothelial cells (HUVEC).^21^ A lectin‐like oxidized low‐density lipoprotein receptor (LOX‐1) is localized to endothelial cells,^22^ where they mediate many biological effects of OxLDL in these cells.^23^ Moreover, OxLDL can shorten endothelial progenitor cells (EPC) survival and impair their functionality in vitro by an inhibitory effect on endothelial NO synthase (eNOS).^24^ Similarly, Chen and colleagues reported that high glucose (HG) levels impair the proliferation and function, and reduce the number of EPCs.^17^ Noteworthy, NO donor but not antioxidants reversed the impairments, suggesting the role of NO‐related rather than oxidative stress–mediated mechanisms in hyperglycaemia‐caused EPC down‐regulation. NO has an essential role in altering the cellular oxidative stress via modifying cysteine residues on Keap‐1, which results in dissociation of the transcription factor Nrf2. The free Nrf2 translocates to the nucleus, where it binds to the *antioxidant response element* (ARE) and induces numerous antioxidant enzymes.^25^, ^26^

In light of these findings, we have been suggested that accelerated atherosclerosis develops in DM patients because the combination of hyperglycaemia and OxLDL exerts a synergistic or additive effect on endothelial cells, rendering them dysfunctional and incapable of maintaining vascular tone. Therefore, the current study was designed to investigate the mechanisms underlying combined HG and hyperlipidaemia‐induced ED by using both HUVEC and HMEC‐1 (in vitro) and LDL knockout mice CByJ.129S7(B6)‐Ldlrtm1Her/J (LDLR^−/−^) (in vivo) approaches.

## 2 MATERIALS AND METHODS

### 2.1 In vitro protocols

This protocol was designed to investigate the impact of HG or OxLDL alone or combined on the vitality of HUVECs and HMEC‐1 and their effects on the status of the NO and ET‐1 systems and oxidative burden.

#### 2.1.1 HUVEC and HMEC‐1 culture

Human umbilical vein endothelial cells (HUVEC) and Human microvascular endothelial cell line (HMEC‐1) were cultured in DMEM and MCDB 131 medium, respectively, in the presence or absence of incremental doses of glucose, OxLDL alone or combined. Specifically, incremental doses of glucose (5, 12.5, 25 or 40 mM) in 10% foetal calf serum (FCS) medium for 4 days (medium changed every 24 hours) were added to HUVECs and HMEC‐1 cells. Then, increasing doses of OxLDL (20, 50 or 100 µg/mL) were added for 24 hours. On day 5, the medium was collected every day and stored at −80°C for ET‐1 and NO metabolites determination as described below. Cells were washed three times with PBS; then, radioimmunoprecipitation assay buffer (RIPA buffer) [150 mmol/L sodium chloride, 1% NP‐40, 0.5% sodium deoxycholate, 0.1% sodium dodecyl sulphate, 50 mmol/L Tris pH 8.0, with protease inhibitor (Complete Mini EDTA‐free protease inhibitor cocktail; Roche, Mannheim, Germany Cat# 11873580001)] was added to the cells, incubated at 4°C for 30 minutes. Following this, the liquid was collected and centrifuged at 13 000 RPM for 10 minutes. The supernatant was collected, and protein concentrations were estimated using Bradford reagent (Cat# B6916, Sigma Aldrich) with Bovine Serum Albumin (BSA) as standard.

The impact of hyperglycaemia and hyperlipidaemia on cells viability, the status of nitric oxide (NO) and endothelin (ET)‐1 systems as well as their signal transduction in cultured HUVEC and HMEC‐1 was assessed using MTT, ELISA, Western blot and immunoreactive staining techniques as detailed hereby:

#### 2.1.2 Cell viability

WST‐8 reagent solution (10 µL) was added to each well containing 100 µL of cells in the culture medium, and the plate incubated for 3 hours at 37°C. Subsequently, DMSO (100 µL) was added to dissolve the resulting formazan by sonication. Absorbance of each well was measured at 570 nm using a microplate reader. Cell survival was expressed as the percentage of MTT formazan absorbance.

##### Measurement of ET‐1 and NO in the medium of cultured HUVEC and HMEC‐1

The intracellular activity of ET and eNOS systems of cultured HUVEC and HMEC‐1 was determined via the amount of ET‐1 and NO in the culture medium. ET‐1 concentration in the medium samples was determined by commercial ELISA (Cayman, Ann Arbor, Michigan, USA). In addition, NO metabolites (NO_2_ + NO_3_) content in the medium was measured by ELISA (Calbiochem, Darmstadt, Germany). NO metabolites in HMEC‐1 were measured by colorimetric assay (Cayman, Ann Arbor, Michigan, USA).

##### Measurement of ECE, ETA, ETB, eNOS and Nrf2 abundance in HUVEC and HMEC‐1

Protein extracts of HUVEC and HMEC‐1 cells that were exposed to HG or OxLDL alone or in combination were prepared by RIPA lysis buffer (Millipore) containing protease and phosphatase inhibitor. The cell lysates were first resolved on SDS‐PAGE gels and transferred to polyvinyldene difluoride membranes by electroblotting. Membranes were incubated with 1:500 diluted monoclonal antibodies against eNOS, phosphorylated eNOS (p‐eNOS) (Santa Cruz), ECE, ETA, ETB, Nrf2, pNrf2 and CD31 (Abcam) in PBS for one hour at room temperature. The membranes were then washed thoroughly in Tris‐buffered saline contained 0.1% (v/v) Tween 20 before a second incubation for one hour at room temperature with a 1:1000 diluted horseradish peroxidase‐conjugated secondary antibody (Santa Cruz). An antibody against β‐actin (Santa Cruz) was used to normalize protein loading. The resultant bands were quantified by densitometry.

##### Effects of HG or OxLDL on cell migration

Migration of HMEC‐1 was evaluated using IncuCyte live image system (Essen BioScience). Cells were grown in image lock 96‐well plate (Essen BioScience Cat# 4379) in MCDB‐131 medium either in normal glucose (5.5 mmol/L) or high glucose (25 mmol/L) for 3 days. Cells were treated with OxLDL (50 µg/mL) for additional 24 hours. Scratch was initiated by an automated scratcher at 100% confluence on day 5. Cells were incubated for additional 24 hours in IncuCyte ZOOM^®^ system. Phase‐contrast images of wounded areas were taken every 1 hour, two images for each well, n = 5 of each treatment (NG, HG, OxLDL and Combination). The system provided calculation of cell density in the wound area expressed relative to the cell density outside of the wound area over the time.

### 2.2 In vivo protocols

**Figure 1:**
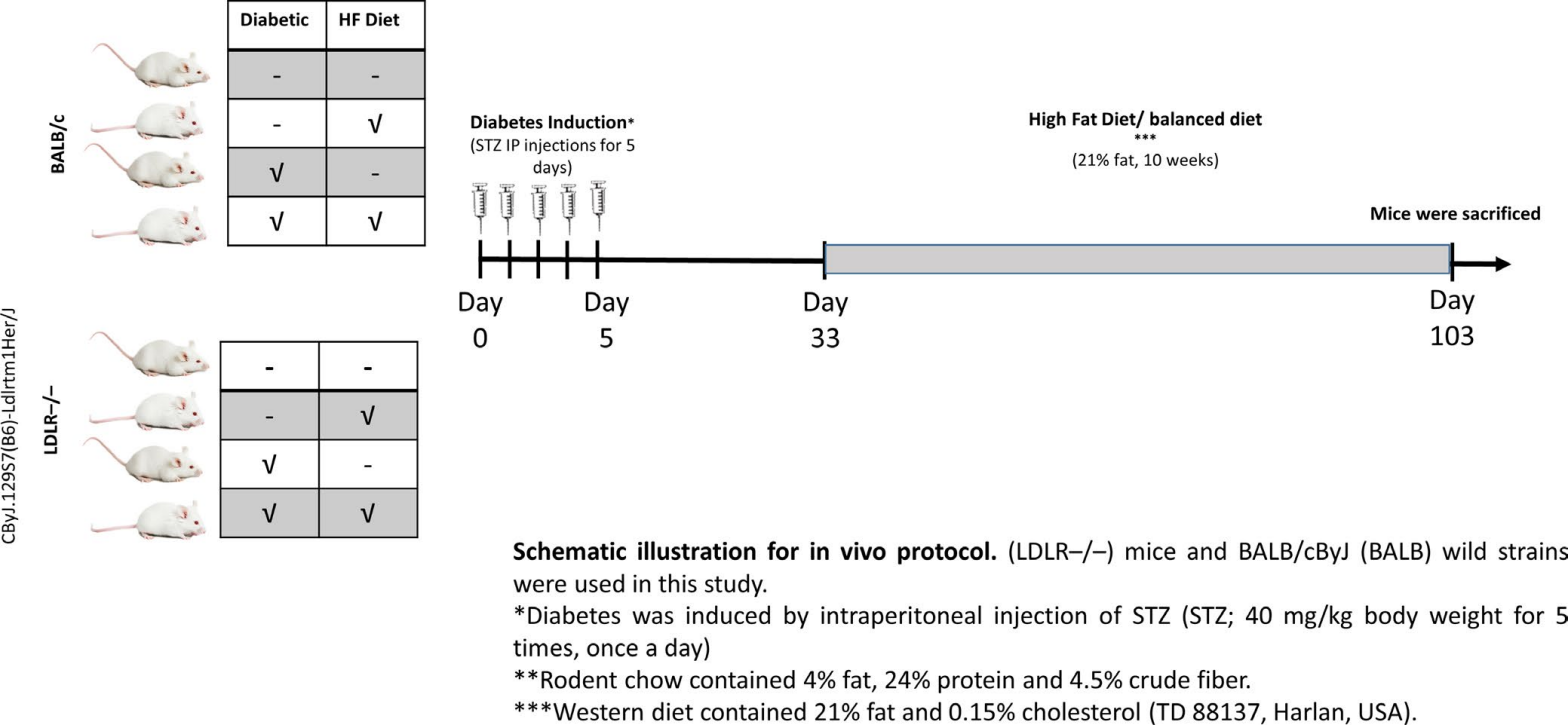
Description of the experimental groups that were included in the in vivo studies. BALB/c mice (n = 20 in total 5 each group) and LDL knockout mice CByJ.129S7(B6)‐Ldlrtm1Her/J (LDLR^−/−^) (n = 20 in total 5 each group) were fed with either standard mouse chow (4% fat, 24% protein and 4.5% crude fibre) or high‐fat diet (21% fat and 0.15% cholesterol [TD 88137, Harlan, USA]) and tap water ad libitum. DM was induced in both BALB/c and LDLR^−/−^ by the injection of (STZ; 40 mg/kg bodyweight five times, once a day) dissolved in sodium citrate buffer (pH 4.5). Animals that were injected with saline served as non‐diabetic corresponding controls. The animals were maintained on normal chow for 4 wk. Then, WT and LDLR^−/−^ were divided into two subgroups, the first group was fed with high‐fat diet for 10 wk, while the 2nd group was placed on regular rodent diet. At the end of the experiment, mice were killed and blood samples were collected

This protocol was designed to examine whether our findings in protocol A (in vitro) persists also in in vivo system consisted of hyperglycaemic, hyperlipidaemic and combined hyperglycaemic and hyperlipidaemic mice and its influence on the status of the NO, ET‐1 system and oxidative stress parameters (Figure 1).

#### 2.2.1 Animals

Studies utilized wild‐type (WT) BALB/c mice (n = 20) and LDL knockout mice CByJ.129S7(B6)‐Ldlrtm1Her/J (LDLR^−/−^) (n = 20) (The Jackson Laboratories). The CByJ.129S7(B6)‐Ldlrtm1Her/J mice are homozygous for the knock out (KO) in exon 4 of the low‐density lipoprotein receptor (Ldlr) gene. The mice are fertile, have normal size and normal physical behavioural abilities. On regular balanced diet, the mice have increased cholesterol (~200 mg/dL), increased triglycerides (~170 mg/dL) and increased blood glucose (122 mg/dL) as compared with wild‐type (WT). For more information visit the following link https://www.jax.org/strain/012845.

Animals were fed with either standard mouse chow (4% fat, 24% protein and 4.5% crude fibre) or high‐fat diet (21% fat and 0.15% cholesterol [TD 88137, Harlan, USA]) and tap water ad libitum. All experiments were approved and performed according to the Technion's guidelines of the Committee for the Supervision of Animal Experiments.

### 2.3 Induction of diabetes

Diabetes mellitus was induced in both BALB/c and CByJ.129S7(B6)‐Ldlrtm1Her/J (LDLR^−/−^) by streptozotocin (STZ) injection (40 mg/kg bodyweight five times, once a day, Sigma Aldrich) dissolved in sodium citrate buffer (pH 4.5). Diabetes was confirmed 5 days later by blood glucose sampling from the tail tip with a glucometer (Ascensia Elite XL, Milan, Italy). Animals that were injected with 0.9% saline served as non‐diabetic corresponding controls. The animals were maintained on normal chow for 4 weeks. Then, WT and LDLR^−/−^ were divided into two subgroups, the first group was fed with high‐fat diet (n = 5) for 10 weeks, while the 2nd was placed on regular rodent diet. We had eight experimental subgroups (Figure 1):

1. BALB/c non‐diabetic‐Normal chow.
2. BALB/c non‐diabetic‐High‐fat diet.
3. BALB/c diabetic‐Normal chow.
4. BALB/c diabetic‐High‐fat diet.
5. LDLR^−/−^ non‐diabetic‐Normal chow.
6. LDLR^−/−^ non‐diabetic‐High‐fat diet.
7. LDLR^−/−^ diabetic‐Normal chow.
8. LDLR^−/−^ diabetic‐High‐fat diet.

At the end of the experiment, mice were killed, blood samples were collected and renal and cardiac tissues harvested for biochemical and histological analysis, respectively. Plasma levels of ET‐1 and NO metabolites were measured by using commercial ELISA kits (Cayman and Calbiochem, respectively).

### 2.4 Statistical analysis

Data are expressed as the mean value or percentage ± standard error of the mean (SEM). Comparisons between the various treatment groups were performed by one‐way analysis of variance with a Bonferroni test for multiple comparisons. Differences were considered to be statistically significant when *P* ≤ .05. Data analysis was performed using a computerized statistical software package (GraphPad Prism version 5.0).

## 3 RESULTS

Based on the findings from our pilot studies (data not shown) and taking into consideration the common level of glucose in uncontrolled hyperglycaemic patients, we chose to examine the effects of 25 mmol/L glucose in combination with or without OxLDL (50 µg/mL) on the status of NO, ET, and oxidative stress systems in cultured HUVEC and HMEC‐1.

### 3.1 In vitro experiments

**Figure 2:**
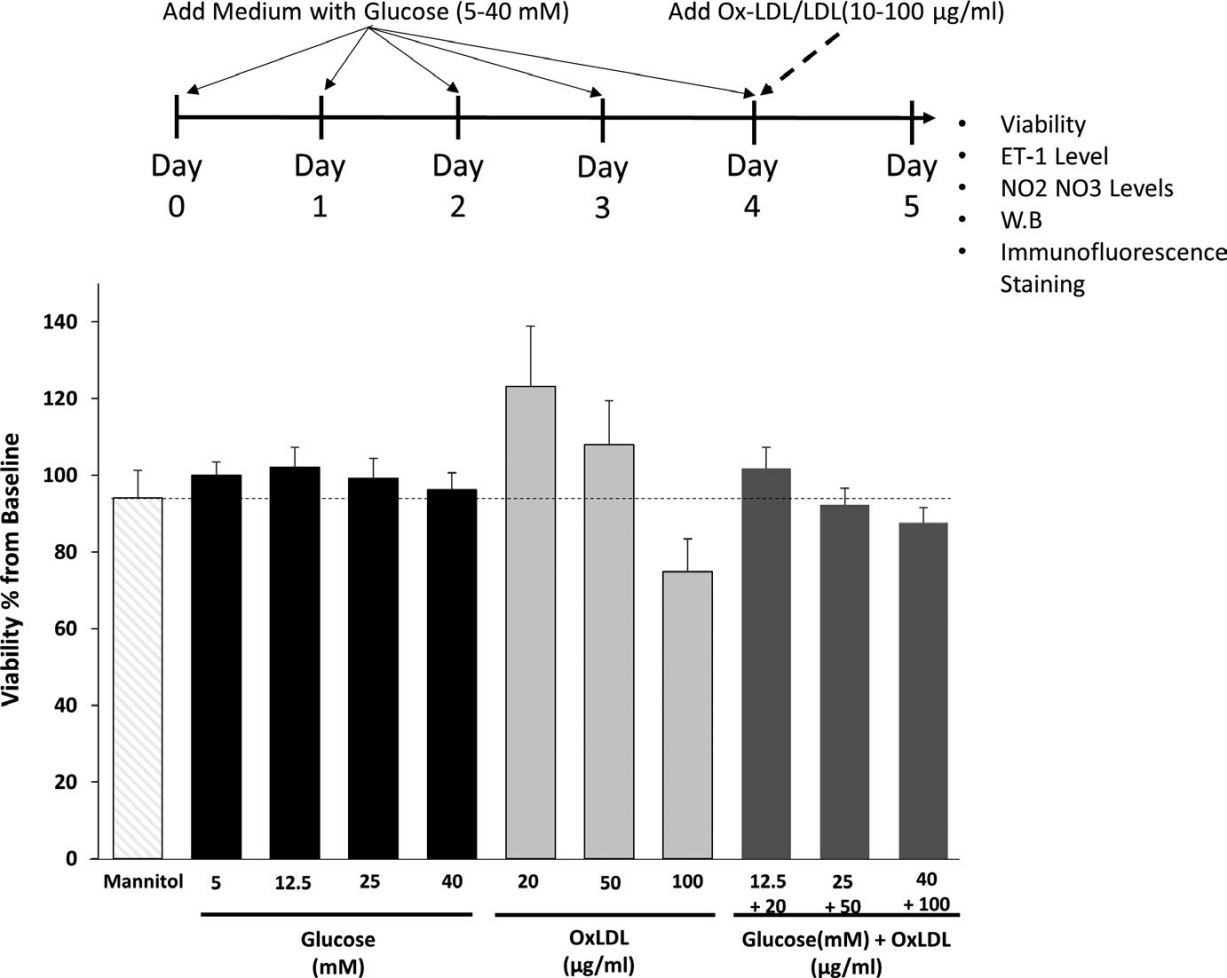
The impact of HG or OxLDL alone or combined on the vitality of HUVECs and HMEC‐1 cells. HUVECs were cultured in DMEM, supplemented with 10% FCS, when reached ~ 70% confluence, cells were grown in 5, 12.5, 25, 40 mmol/L glucose for 4 d with daily medium replacement. On day 4, cells were treated with 20, 50 and 100 μg/mL of OxLDL with different concentration of glucose for additional 24 h. Cell viability was assessed on day 5 using WST‐8 reagent, and cell survival was expressed as the percentage of MTT formazan absorbance of the 5.5 mmol/L glucose, 0 μg/mL of OxLDL treated cells. The results are represented as the mean ± SEM of 3‐5 independent experiments with triplicates in each

The viability of HUVECs was not impacted by high glucose levels (up to 40 mmol/L for 4 days) or OxLDL (up to 100 µg/mL for 24 hours), indicating that these concentrations are not toxic in the given time frames. Moreover, the combinations of glucose and OxLDL had minor impact on cell viability as compared to cells cultured in 5 mmol/L glucose (Figure 2). Mannitol was used to roll out the impact of osmolality, where 25 mmol/L mannitol has no impact on HUVECs viability.

### 3.2 Effects of HG or OxLDL on the endothelin system of cultured HUVEC and HMEC‐1

**Figure 3:**
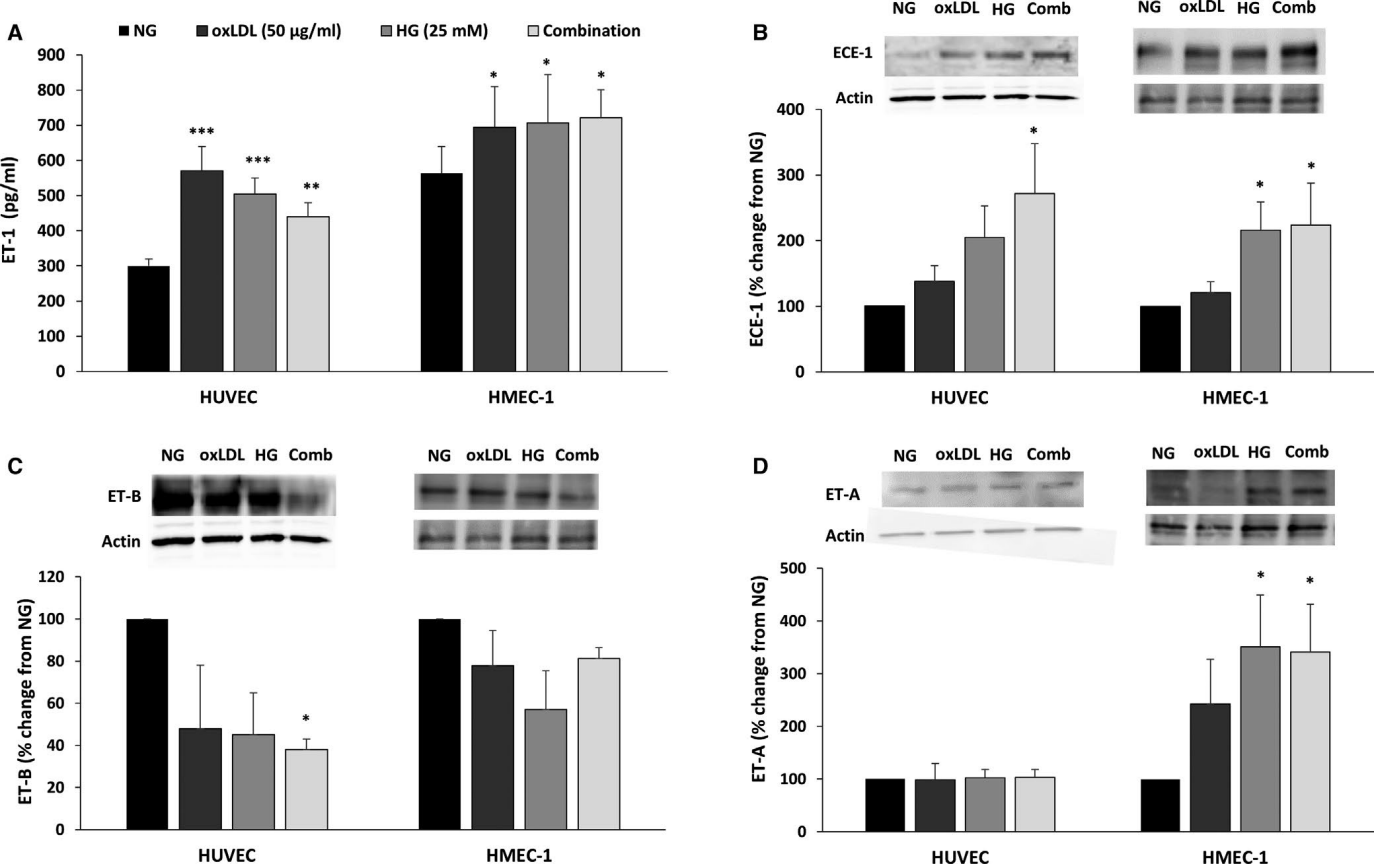
High glucose (HG), OxLDL and combination of both increase ET‐1 levels in HUVECs and HMEC‐1 cells. HUVECs and HMEC‐1 cells were cultured in DMEM and MCDB 131 medium, respectively, supplemented with 10% FCS. When reached ~ 70% confluence, cells were grown in 5.5 mmol/L glucose considered as normal (NG), or 25 mmol/L considered as HG, for 4 d with daily medium replacement. On day 4, cells were treated with or without 50 μg/mL of oxidized LDL (OxLDL) for additional 24 h with or without the indicated glucose concentrations. Following collection of the cells and supernatants (day 5), ET‐1 secretion in was measured using ELISA (A). ECE‐1 (B), ET‐B (C) and ET‐A (D) expressions were measured in Western blot and quantified in densitometry analysis. Results are represented as mean ± SEM, n = 4‐12 in each treatment group. *P < .05, **P < .01, ***P < .001

Figure 3 depicts the impact of high dose of glucose, OxLDL or combination of both on ET‐1 secretion to the media, ECE‐1, a key enzyme of ET‐1 production, ET_B_, a vasodilatory ET‐1 receptor subtype, and ET_A_, a vasoconstrictor ET‐1 receptor subtype in cultured HUVEC and HMEC‐1. HG treatment significantly increased the secretion of ET‐1 in both cell lines (1.68‐fold and 1.25‐fold increase for HUVECS and HMEC‐1, respectively). Similarly, OxLDL treatment significantly enhanced the secretion of ET‐1 (1.9‐fold and 1.23‐fold increase for HUVECS and HMEC‐1, respectively) (Figure 3A). However, the combination of HG and OxLDL did not induce additive effect on ET‐1 secretion. Mannitol was used to roll out the impact of osmolality of HG on HUVEC cells. Figure S1 shows that 25 mmol/L mannitol has no impact on endothelin and nitric oxide metabolites secretion, suggesting that the impact of glucose is osmolality independent.

ECE‐1 immunoreactive levels were significantly increased upon the addition of either HG or OxLDL to HUVECs (2.05‐fold and 1.3‐fold increase, respectively). Similar trend was obtained when HG or OxLDL were added to HMEC‐1 (2.1‐fold and 1.2‐fold increase following HG or oxLDL treatment, respectively). Combination of HG and OxLDL yielded an additive effect on ECE‐1 abundance in both cell types (2.7‐fold and 2.4‐fold increase in HUVECs and HMEC‐1, respectively) (Figure 3B).

The stimulatory effects of HG, OxLDL alone or combined on ET‐1 secretion and ECE abundance, immunoreactive levels of ET_B_ in HUVEC were diminished significantly when both HG and OxLDL were added (~60% reduction) (Figure 3C). In contrast, ET_A_ abundance was increased by combination of HG and OxLDL in HMEC‐1 cells (3.4‐fold increase), yet was not affected in HUVECS (Figure 3D).

### 3.3 Effects of HG or OxLDL on nitric oxide (NO) system in cultured HUVEC and HMEC‐1

**Figure 4:**
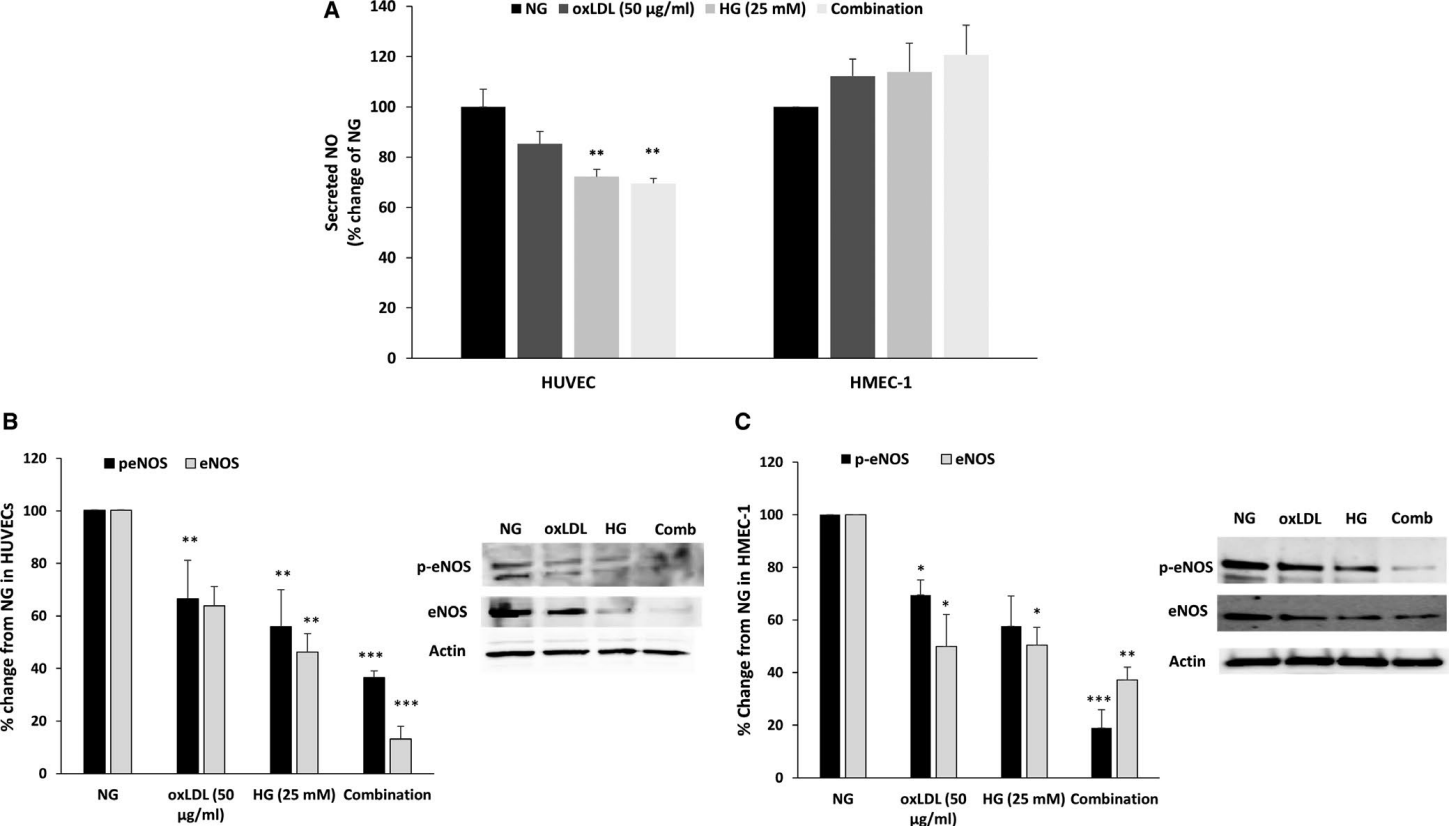
Combination of HG and OxLDL significantly down‐regulates eNOS activity. HUVECs and HMEC‐1 were cultured and treated as in Figure 3. NO metabolites secretion in the supernatants was measured using colorimetric assay (A). HUVECs (B) and HMEC‐1 (C) cells were collected and examined for eNOS and p‐eNOS expression using Western blot. Results are represented as mean ± SEM, n = 2‐9 in each treatment group. *P < .05, **P < .01, ***P < .001

In contrast to the stimulatory effect of HG and OxLDL on ET‐1 secretion, these substances exerted inhibitory effects on nitrite (NO_2_) and nitrate (NO_3)_ secretion of HUVECs, especially when both HG and OxLDL were added simultaneously (~25% reduction) (Figure 4A). In line with these findings, the addition of HG or OxLDL alone or combined to either HUVEC and HMEC‐1 induced down‐regulation of both eNOS (90% and 63% reduction following combination treatment) and phosphorylated eNOS (peNOS) the active isoform of this key enzyme in NO production by endothelial cells (62% and 82% reduction following combination treatment for HUVECs and HMEC‐1, respectively) (Figure 4B,C).

### 3.4 Effects of HG or OxLDL on Nrf2 abundance in cultured HUVEC

**Figure 5:**
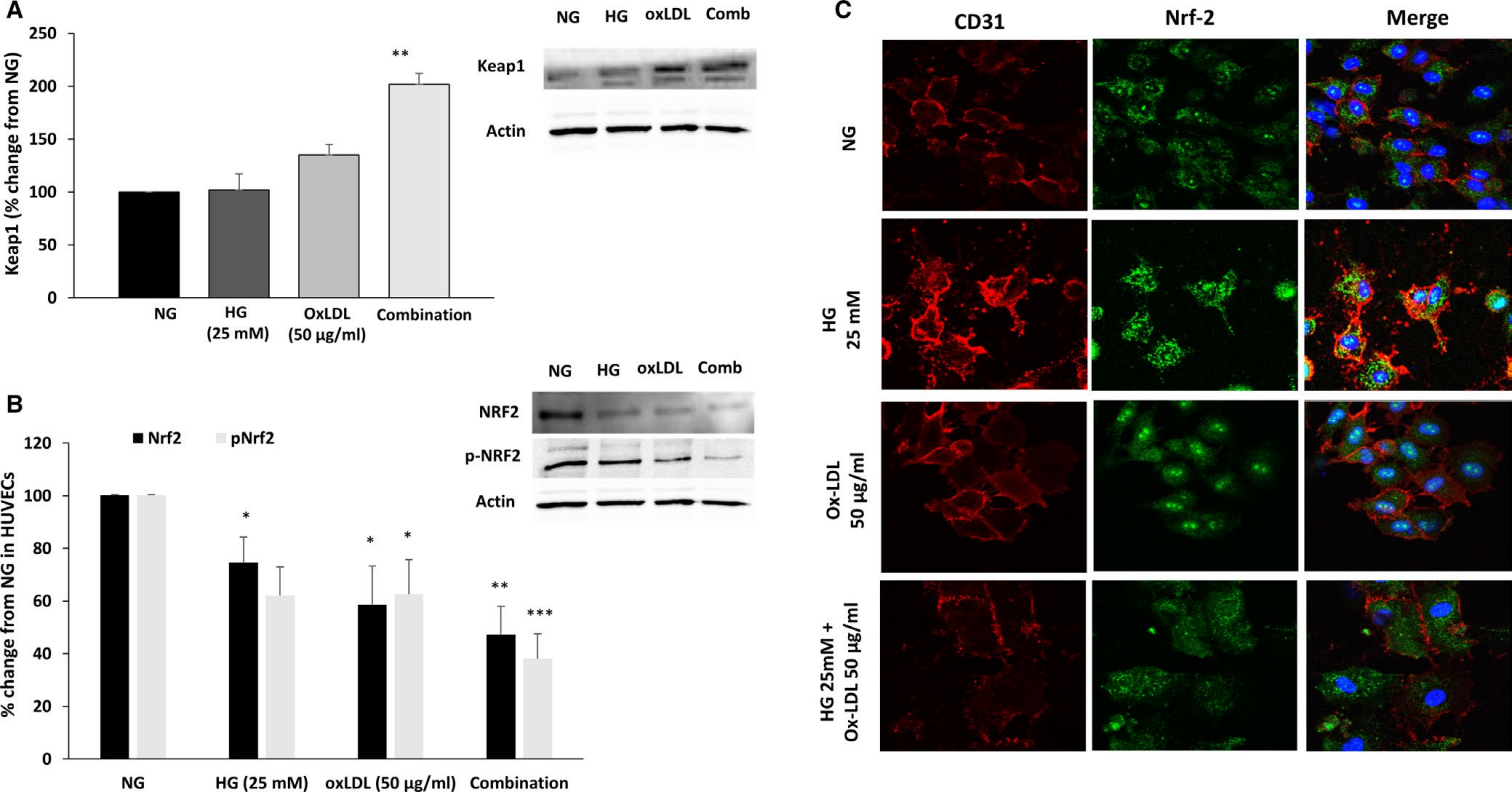
Combination of HG and OxLDL decreases Nrf2 levels, activity and translocation in the nucleus. HUVECs were cultured and treated as in Figure 3, then examined for Keap1 expression (A) as well as Nrf2 expression and phosphorylation (B) in Western blot and quantified in densitometry analysis. Endothelial marker CD31 (red) and Nrf2 (green) immunofluorescence were examined following the indicated treatments (C). Results are represented as mean ± SEM, n = 4 in each treatment group. *P < .05, **P < .01, ***P < .001

The current protocol was designed to explore the downstream connection between NO and Nrf2 in HUVEC exposed to HG and OxLDL. Keap‐1 immunoreactive levels were increased upon the addition of either HG or OxLDL to HUVECs. Combination of HG and OxLDL yielded an additive effect on Keap‐1 abundance (~twofold increase) (Figure 5A). In contrast, phosphorylated and non‐phosphorylated Nrf2 immunoreactive proteins were decreased by ~ 50% in HUVEC, especially when HG and OxLDL were simultaneously added (Figure 5B). However, the effect of HG and OxLDL on Keap 1 and Nrf2 expression was not consistent in HMEC‐1 (data not shown).

The immunostaining intensity of total Nrf2 (green staining panel in Figure 5C) was not significantly affected by OxLDL (50 µg/mL) or HG (25 mmol/L). However, Nrf2 activation and translocation to the nucleus increased when the cells were incubated with either OxLDL for 24 hours or HG for five days, which is shown in the merged panel as green dots on the nucleus blue area. Interestingly, Nrf2 translocation to the nucleus was abolished when 50 µg/mL OxLDL were added for 24 hours on cells cultured with 25mM HG for five days (Figure 5C).

### 3.5 Effects of HG or OxLDL on HMEC‐1 migration

**Figure 6:**
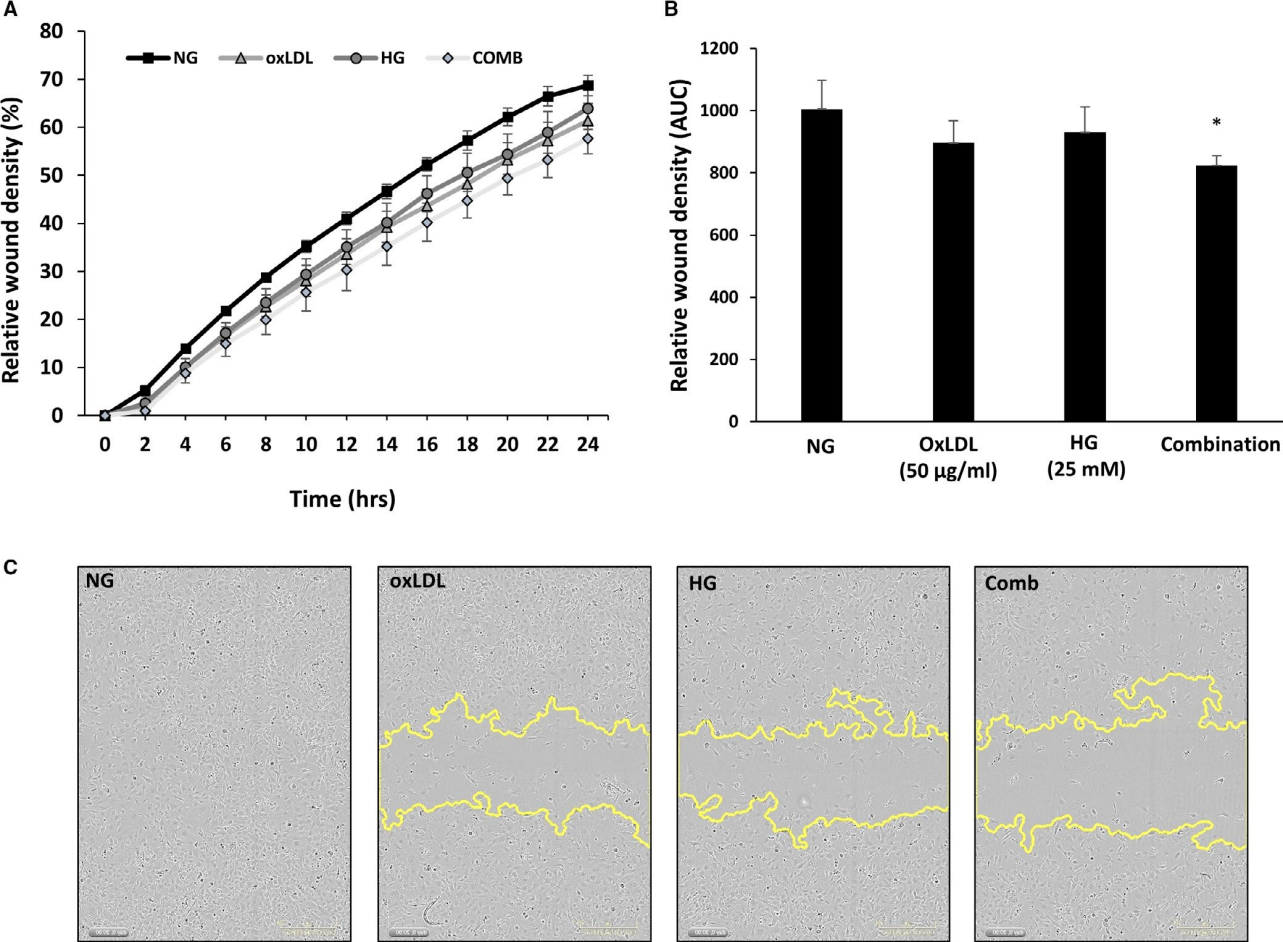
High glucose (HG), OxLDL and combination of both decreases endothelial cell migration in scratch wound assay. HMEC‐1 cells were incubated with either HG or NG for 4 d and with or without OxLDL (50 µg/mL) for additional 24 h. Then, cells were wounded by automated scratcher at 100% confluence. Medium was switched from 10% FCS to starvation mode (2% FCS) 2‐3 h prior to scratch performance and throughout scratch evaluation. Phase‐contrast images of wounded areas were taken each hour over a period of 24 h using IncuCyte ZOOM^®^ system. A, Relative wound density of the wound of each group over 24 h. B, Area under the curve of the wound density. C, Representative images of the wound of each cell type and each treatment at 24 h. *P < .05

Endothelial cells migrate during vasculogenesis and angiogenesis but also in a damaged vessel to restore vessel integrity. Endothelial cell migration ability following treatment with HG, OxLDL or combination of both was evaluated using the wound healing scratch assay. As shown in Figure 6, OxLDL and HG treatment alone slightly decreased the relative wound density of HMEC‐1 cells compared to cells incubated in normal glucose conditions (10.8% and 7.4% reduction), respectively. A statistically significant additional reduction of 18.1% in relative wound density was observed in cells that were treated with combination of HG and OxLDL.

### 3.6 In vivo experiments

**Figure 7:**
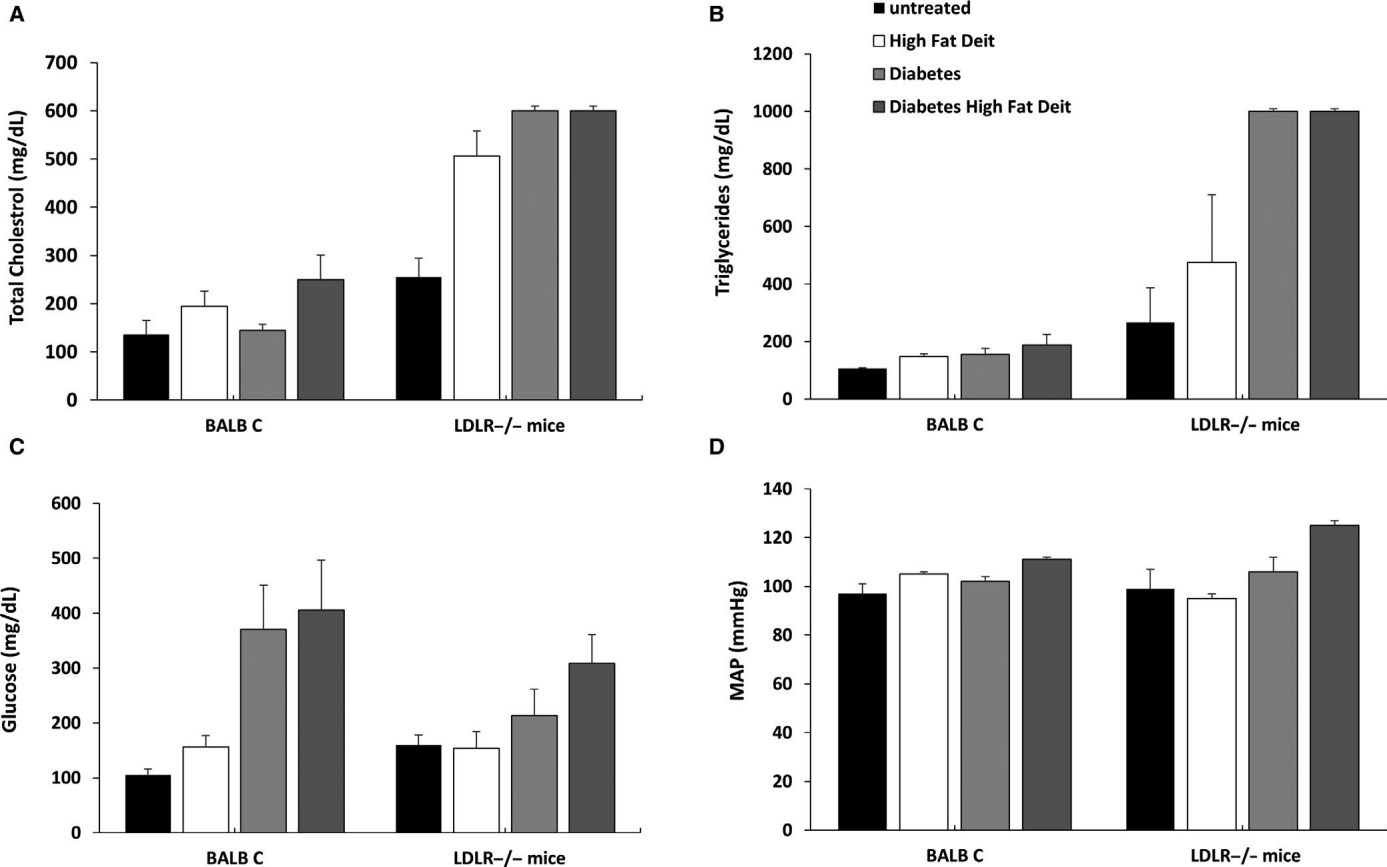
Effect of HF chow and DM alone or combined on various biochemical and physiological parameters Balb C mice and LDLR^−/−^ animals. Both LDL knockout mice CByJ.129S7(B6)‐Ldlrtm1Her/J (LDLR^−/−^) and their wild‐type (WT) BALB/c mice were fed with either standard mouse chow (4% fat, 24% protein and 4.5% crude fibre) or high‐fat diet (21% fat and 0.15% cholesterol). DM was induced in both subgroups and maintained on normal chow for 4 wk. Then, WT and LDLR^−/−^ were divided into two subgroups, the first group was fed with high‐fat diet (n = 5) for 10 wk, while the 2nd was placed on regular rodent diet. At the end of follow‐up period and after BP measurement, the animals were killed and blood specimens were collected for biochemical and molecular analysis. A, Cholesterol levels. B, Triglyceride levels. C, Glucose levels. D, Mean arterial blood pressure (MAP) in Balb C mice and LDLR^−/−^ animals. Results are expressed as Mean + SEM

This protocol was carried out to assess the impact of diabetes, high‐fat (HF) diet or combination of both on ET‐1 and NO systems in diabetic LDLR^−/−^ mice and their wild‐type controls, Balb C mice. HF chow increased plasma cholesterol, but not triglycerides (TG) levels in Balb C mice. Basal levels of cholesterol and TG were higher in LDLR knockout mice. When the latter were placed on HF chow, increased levels of cholesterol and triglycerides were obtained (Figure 7). While induction of diabetes in Balb C did not affect the cholesterol and TG levels, combination of HF and diabetes in these mice yielded a further yet moderate increase in these parameters. In contrast, LDLR^−/−^ mice exhibited substantial increase in cholesterol and TG when fed with HF, exposed to DM, and preferentially when DM + HF were combined (Figure 7).

As presented in Figure 7, STZ‐induced DM in Balb C mice resulted in higher glucose levels mainly when combined with HF chow. Glucose levels were also increased in LDLR^−/−^ following STZ‐induced DM, mainly when combined with HF chow (Figure 7). DM, high‐fat chow or combination of both increased blood pressure (BP) in BALB, whereas mean arterial blood pressure (MAP) increased in LDLR^−/−^ mice only when HF was combined with DM (Figure 7). However, kidney and liver function as well as histological features were normal in the different mice groups during the study timeline (data not shown). In addition, we measured kidney and heart weight normalized to bodyweight as well as we performed histological analysis of the kidney (Figure S2). No significant changes in kidney and heart weight nor histological alterations in the renal tissue were observed among the various experimental groups (Figure S2).

**Figure 8:**
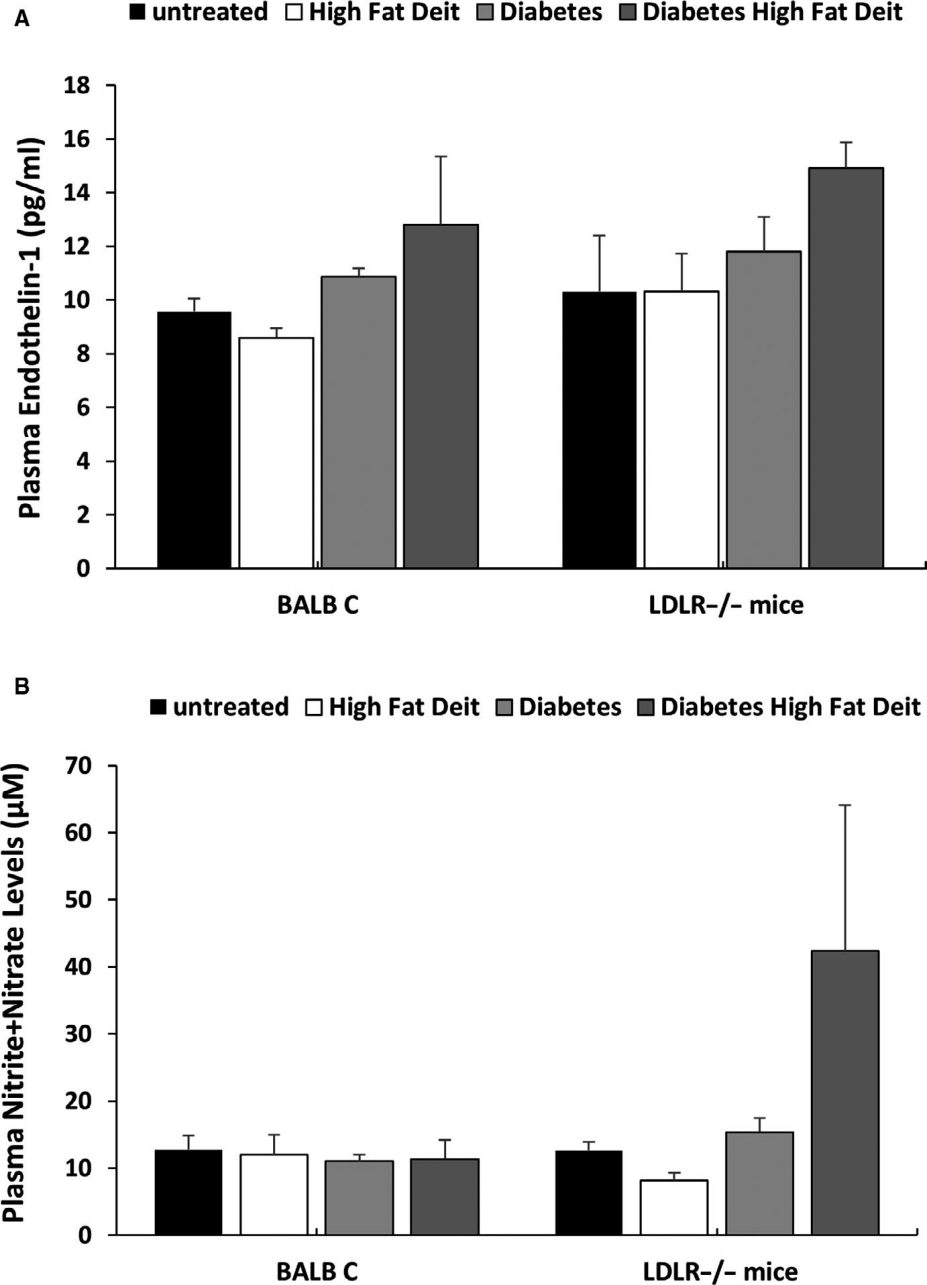
Effect of HF chow and DM alone or combined on circulatory levels of ET‐1 and NO metabolites in Balb C mice and LDLR^−/−^ animals. Both LDL knockout mice CByJ.129S7(B6)‐Ldlrtm1Her/J (LDLR^−/−^) and their wild‐type (WT) BALB/c mice were fed with either standard mouse chow (4% fat, 24% protein and 4.5% crude fibre) or high‐fat diet (21% fat and 0.15% cholesterol. DM was induced in both subgroups and maintained on normal chow for 4 wk. Then, WT and LDLR^−/−^ were divided into two subgroups, the first group was fed with high‐fat diet (n = 5) for 10 wk, while the 2nd was placed on regular rodent diet. At the end of follow‐up period, the animals were killed and blood samples were collected for measurement of circulating ET‐1 and NO metabolites (NO2 + NO3). A, Cholesterol levels. B, Triglyceride levels. C, Glucose levels. D, Mean arterial blood pressure (MAP) in Balb C mice and LDLR^−/−^ animals. (A) and NO2 + NO3 (B) in Balb C mice (n = 5 each group and LDLR^−/−^ animals). Results are expressed as Mean + SEM

Neither HF chow nor DM affects plasma ET‐1 in both Balb C and LDLR knockout mice (Figure 8). Interestingly, combination of DM and HF elevated blood ET‐1 in both Balb C and LDLR^−/−^by 34% and 45%, respectively (Figure 8A). HF chow or DM did not influence plasma NO metabolites neither in Balb C nor in LDLR knockout mice. Stimulatingly, combination of DM and HF caused a remarkable increase in NO levels in LDLR^−/−^ animals (~3.4‐fold increase) (Figure 8B).

## 4 DISCUSSION

The present study provides new insights into the mechanisms underlying hyperglycaemia and hyperlipidaemia‐induced ED. Our findings clearly show that exposure of either HUVEC or HMEC‐1 cells to HG or OxLDL induced ET‐1 production. Overproduction of ET‐1 probably stem from up‐regulation of ECE‐1 as observed under these conditions. In contrast, combination of HG and OxLDL dramatically decreased peNOS in HUVECs and HMEC‐1. Likewise, ETB levels on both types of endothelial cells significantly declined from baseline following exposure to HG or OxLDL alone or combined. Moreover, combined HG and OxLDL decreased Nrf2 (transcription factor for various antioxidant enzymes) as compared to baseline. HG or OxLDL treatment alone was sufficient to decrease the migration rate of HMEC‐1 cells compared to normal glucose conditions. A significant additional reduction in migration rate was observed in cells that were treated with combination of HG and OxLDL. These ‘in vitro*’* findings were supported by ‘in vivo*’* studies, where diabetic LDLR^−/−^ mice displayed higher blood pressure, plasma cholesterol and glucose concentrations, along elevated circulatory ET‐1 and NO_2_/NO_3_ levels, as compared with normoglycemic LDLR^−/−^ and BALB mice. Collectively, these results show that combined diabetes and hyperlipidaemia is characterized by activation of the ET system and attenuated eNOS and Nrf2 level/activity, suggesting that perturbations in these paracrine systems may contribute to ED.

Intact endothelium is essential for vascular function as it participates in regulation of vascular tone, endothelium interaction with blood constituents and blood vessels permeability.^27^ Endothelial dysfunction due to hyperlipidaemia and hyperglycaemia has been documented both in vivo and in vitro.^28^ For instance, OxLDL promotes oxidized lipid accumulation in macrophages and vascular smooth muscle cells (VSMC) in vitro^29^ and induces toxic effect on various cultured cell types such as lymphocytes, monocytes, macrophages, endothelial cells, VSMC and fibroblasts.^30^ Moreover, the diabetic milieu, that is hyperglycaemia and high levels of circulatory free fatty acids provokes oxidative stress and low‐grade inflammatory responses, which attribute to ED.^31^ The latter is characterized by imbalance between vasodilators and vasoconstrictors. Specifically, among the mile stones of diabetes‐ and atherosclerosis‐induced ED is reduced production and bioavailability of NO, along enhanced production of vasoconstrictors and increased generation of ROS.^31^, ^32^ Although the mechanisms underlying these derangements are multifactorial, our results unambiguously demonstrate that hyperglycaemia and hyperlipidaemia per se play a central role in the development of imbalance between the vasodilators and vasoconstrictors under diabetes/atherosclerosis clinical settings. Specifically, high glucose levels or OxLDL alone or in combination enhanced ET‐1 production along impaired NO production by HUVECs and HMEC‐1 cells. The stimulatory effect of HG and OxLDL on ET‐1 generation, along their inhibitory action on NO production, could be attributed to up‐regulation and down‐regulation of ECE and eNOS, respectively. Similarly, Park et al^33^ demonstrated that high glucose increases ET‐1 mRNA and protein expression via activation of protein kinase C (PKC) β and δ isoforms in bovine retinal endothelial cells. In line with these findings, we have previously demonstrated that exposure of EA.hy926 cell line and HUVEC to high glucose concentrations (22.2 mmol/L) increased ECE‐1 immunoreactivity by twofold after 24h and by 20‐fold after 5 days.^34^ This effect is mediated by PKC, which was up‐regulated in response to HG. Support for this notion is derived from the observation that GF109203X, a general PKC inhibitor, abolished the stimulatory impact of HG on ECE expression.^34^ However, not all studies demonstrated stimulatory effect of HG on ET‐1 secretion by endothelial cells, as some studies reported an inhibitory effect or no effect.^35^ These discrepancies may stem from the EC type applied, culturing conditions and differences in concentrations of glucose.

Besides the stimulatory effect of HG on ET‐1 secretion, the current study revealed inhibitory effect of HG on ETB expression in both HUVECs and HMEC‐1 cells, which reached statistical significance only when HG and OxLDL were combined. Of note, ETB mediates the vasodilatory actions of ET‐1 in endothelial cells. Thus, this observation may explain the impaired vasodilation and exaggerated stiffness of blood vessels characterizing diabetes.^4^, ^31^ Support for this assumption is further derived from our observation that either HG or OxLDL alone or combined remarkably decreased both total eNOS, and peNOS. This finding is in line with previous studies that examined the impact of HG or OxLDL alone on eNOS. For example, Ma et al^19^ showed that OxLDL decreased EPCs survival and impaired its adhesive, migratory and tube‐formation capacities in a dose‐dependent manner. Furthermore, OxLDL decreased eNOS expression, indicating that OxLDL inhibits EPC survival and impairs its function, via an inhibitory effect on eNOS. Similarly, Chen et al^17^ demonstrated that incubation with high glucose, but not mannitol, dose‐dependently reduced the number and proliferation of EPCs, and impaired the migration of these cells. These deleterious effects were associated with decreased eNOS and bioavailable NO. Interestingly, the adverse effects of high glucose were ameliorated by co‐incubation with sodium nitroprusside, a NO donor. In agreement with these findings of Ma et al^19^ and Chen et al,^17^ the present study shows that HG and OxLDL alone decreased the migration rate of HMEC‐1 cells compared to normal glucose conditions. A significant additional reduction in migration rate was observed in cells that were treated with combination of HG and OxLDL, suggesting an additive effect of these parameters. Although he hyperglycaemia‐induced reduction in NO metabolites, most likely stems from down‐regulation of eNOS, Brodsky et al ^36^demonstrated that hyperglygemia per se promotes the chemical inactivation of NO. The authors concluded that this glucose‐mediated NO loss may directly contribute to hypertension and ED in diabetic patients.

Similarly, hyperlipidaemia may adversely affect endothelial function, by disrupting the molecular mechanisms regulating NO bioavailability along avid ROS production.^37^, ^38^, ^39^ In this context, our results clearly show that both HG and OxLDL, and largely when combined, reduced endothelial pNrf2 as compared to baseline. Nrf2 is a transcription factor for various antioxidant enzymes. Under normal condition, Nrf2 is bound to Keap 1, maintaining it in inactive form.^26^ Upon appropriate stimuli, such as oxidative stress, diabetes and metabolites, Nrf2 dissociate from Keap 1 and to subsequently translocate into the nucleus, where it binds to the antioxidant response element (ARE) and promotes the transcription of a wide variety of antioxidant genes.^26^ Ungvari et al^40^ demonstrated that HG (10‐30 mmol/L) significantly increases transcriptional activity of Nrf2 and up‐regulates the expression of the Nrf2 in coronary endothelial cells after 24 hours of incubation. Similarly, Afonyushkin et al^25^ reported that OxLDL enhanced the expression of Nrf2 along oxidative stress in cultured in several types of endothelial cells. In agreement with these findings, in vivo models of high‐fat diet Nrf2^−/−^ and Nrf2^+/+^ mice unravelled that a high‐fat diet induced increases in vascular ROS levels were greater in Nrf2^−/−^ than in Nrf2^+/+^ mice.^26^, ^40^ Thus, Nrf2‐Keap1‐ARE pathway represents a compensatory defence mechanism against hyperglycaemia‐ and hyperlipidaemia‐induced oxidative stress. However, the present study demonstrated that combination of OxLDL and HG for five days up‐regulated the immunoreactive levels of Keap 1 along down‐regulation of Nfr2 in HUVECs, but not in HMEC‐1. These findings suggest that incubating the cells with high glucose for five days consume the cells’ antioxidant ability leading to the aberrant behaviour of Nrf2 then promoting the oxidative stress in endothelial cells, thus contributing to ED. Previous in vitro studies in human cells show that NRF2 is activated when cells are exposed acutely to high glucose, yet, longer incubation times fail to activate it. In addition, NRF2 is reported to be down‐regulated in pre‐diabetic and diabetic patients, suggesting that NRF2 is affected not only by the concentration of glucose but also the duration of exposure to high glucose.^41^, ^42^

In order to verify whether similar behaviour exists also at the physiological level, we investigated whether combined HG and hyperlipidaemia‐induced ED in vivo by using LDL knockout mice CByJ.129S7(B6)‐Ldlrtm1Her/J (LDLR^−/−^) subjected to hyperlipidaemia, by high‐fat diet and hyperglycaemia, by inducing DM. In line with the in vitro protocols, diabetic LDLR^−/−^ mice that were placed on high‐fat diet displayed high blood pressure and plasma lipids concentrations along elevated levels of ET‐1 and NO_2_/NO_3_, as compared with normoglycemic LDLR^−/−^ and BALB mice. The elevated levels of ET‐1 most likely derived from the endothelial cells, although we could not exclude other sources of ET producing cell types.^5^ Concerning the high levels of NO_2_ + NO_3_, most likely this elevation does not reflect enhanced activity of eNOS, rather may stim from activation of iNOS, since combined atherosclerosis and diabetes, as the case in diabetic LDLR^−/−^ mice that were placed on high‐fat diet, is characterized by inflammation manifested by cytokines secretion and induction of iNOS.^31^, ^32^

In summary, exposure of cultured endothelial cells to HG and OxLDL is characterized by activation of the ET system and attenuated eNOS and Nrf2 level/activity. Similarly, combined diabetes and hyperlipidaemia in LDL^−/−^ mice induced elevated levels of circulatory ET‐1 along elevated levels of NO metabolites (NO_2_ + NO_3_). These findings suggest that perturbations in these paracrine systems may contribute to ED, a whole mark feature of DM and hyperlipidaemia.

## CONFLICT OF INTEREST

All authors declare that the research was conducted in the absence of any commercial or financial relationships that could be construed as a potential conflict of interest.

## AUTHOR CONTRIBUTION

**Niroz Abu‐Saleh:** Conceptualization (equal); Data curation (lead); Formal analysis (lead); Methodology (lead); Writing‐original draft (equal); Writing‐review & editing (equal). **Hiba Yaseen:** Conceptualization (equal); Data curation (equal); Formal analysis (equal); Methodology (equal); Writing‐original draft (equal); Writing‐review & editing (equal). **Safa Kinaneh:** Methodology (equal); Software (equal). **Mogher Khamaisi:** Conceptualization (equal); Resources (equal); Writing‐original draft (equal); Writing‐review & editing (equal). **Zaid Abassi:** Conceptualization (lead); Data curation (lead); Formal analysis (lead); Funding acquisition (lead); Investigation (lead); Methodology (equal); Project administration (lead); Resources (lead); Software (lead); Supervision (lead); Validation (lead); Visualization (lead); Writing‐original draft (lead); Writing‐review & editing (lead).

## Supporting information

Figure S1

Figure S2

## Data Availability

All data generated or analysed during this study are included in this published article (and its Supporting Information).
