# Supplementary figures and images for: Combination of hyperglycaemia and hyperlipidaemia induces endothelial dysfunction: Role of the endothelin and nitric oxide systems
Source: J Cell Mol Med. 2020 Dec 26;25(4):1884–95. doi: 10.1111/jcmm.15787 (PMC7882960; doi:10.1111/jcmm.15787)

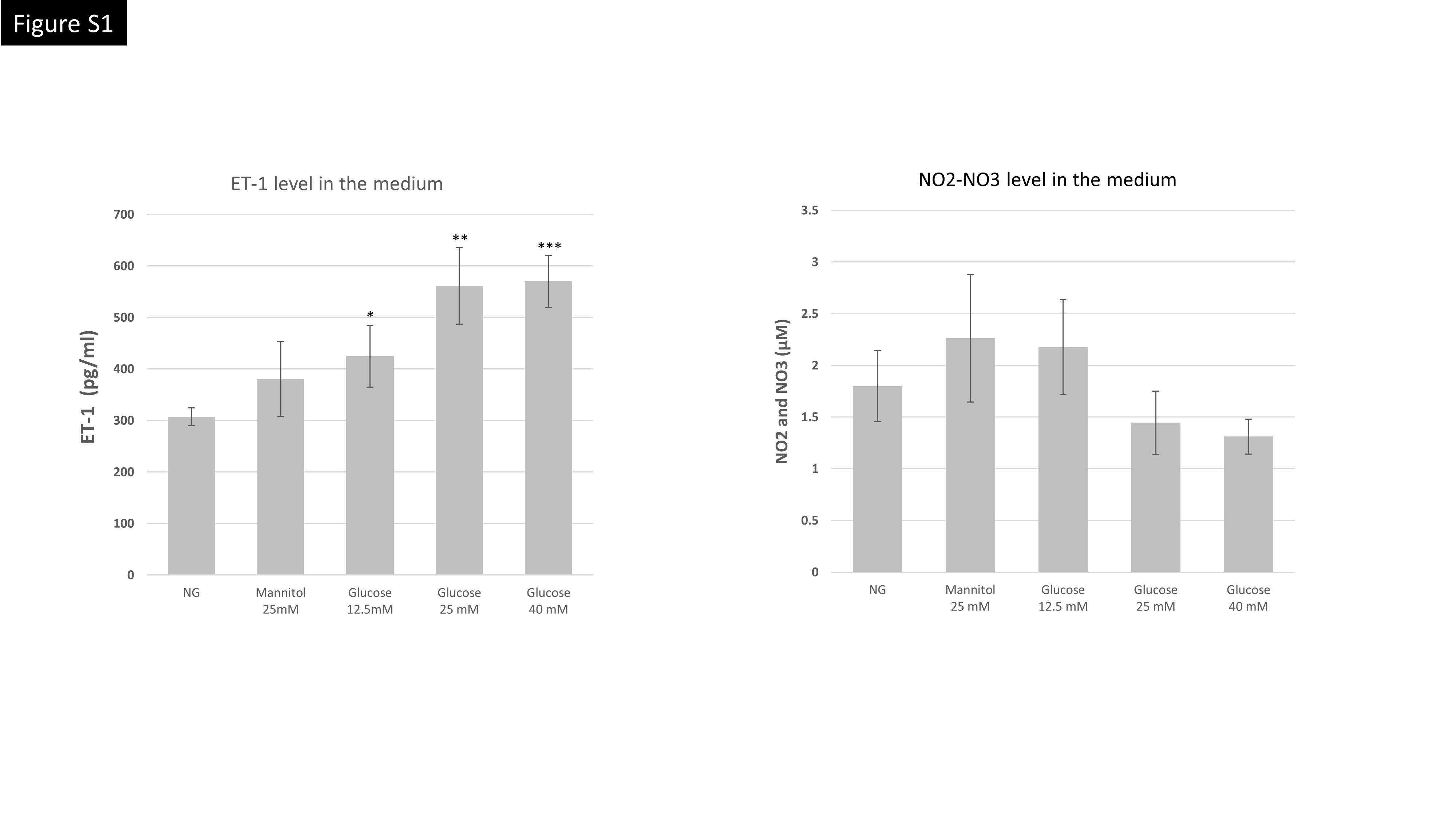

Supplement: Supplementary file 1 — Figure S1 [file JCMM-25-1884-s001.TIF]

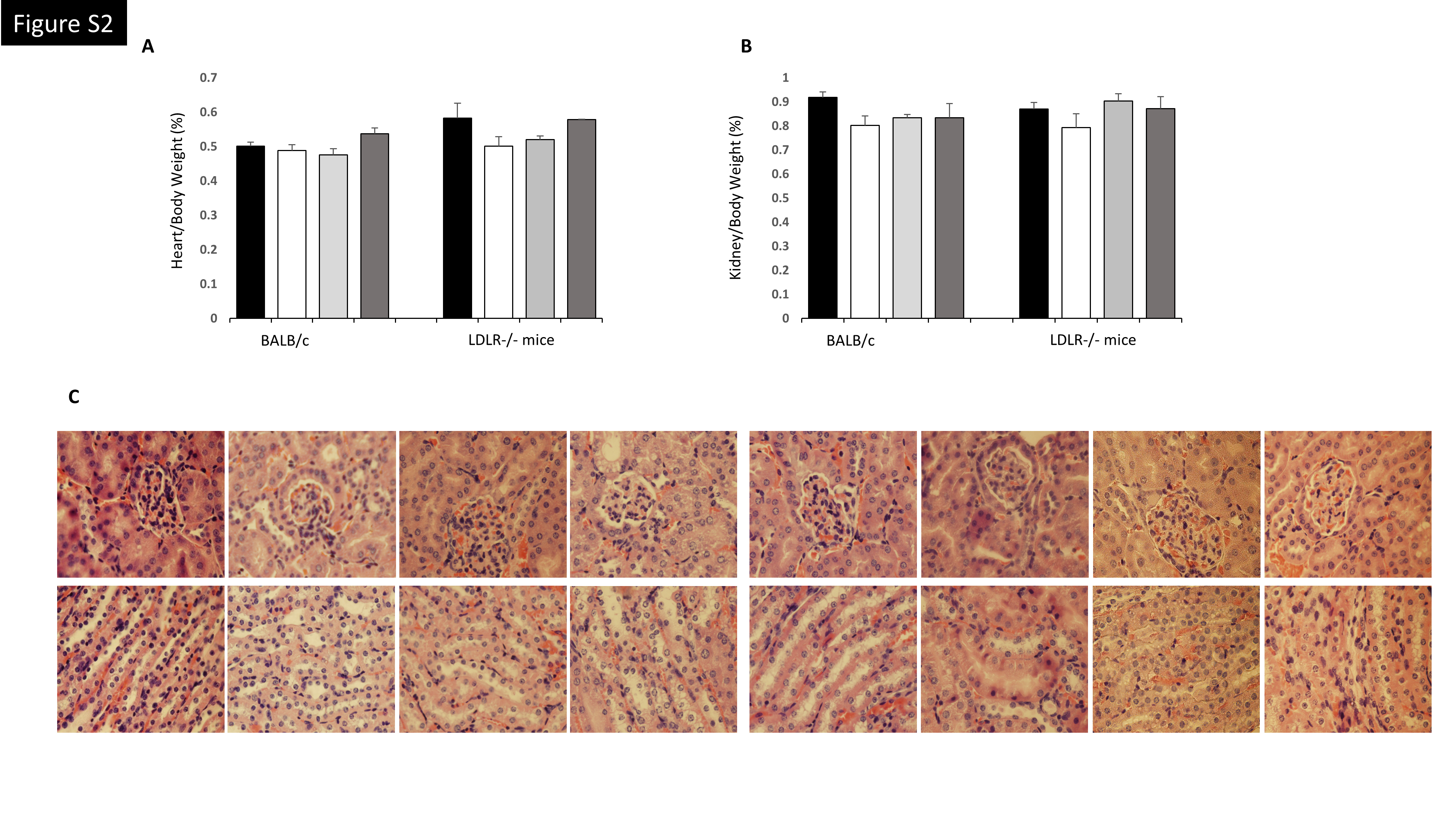

Supplement: Supplementary file 2 — Figure S2 [file JCMM-25-1884-s002.TIF]
